# Extra Hepatic Portal Vein Obstruction with Solitary Left Kidney: A Case Report

**DOI:** 10.31729/jnma.4214

**Published:** 2019-04-30

**Authors:** Pabitra Adhikari, Nirajan Regmi, Akash Chitrakar, Bikal Ghimire, YP Singh

**Affiliations:** 1 Department of GI and General Surgery, Institute of Medicine, Maharajgunj, Kathmandu, Nepal

**Keywords:** *cavernoma*, *modified Hassab's operation*, *Portal vein*, *unilateral renal agenesis*

## Abstract

Extra Hepatic Portal Vein Obstruction in individual with solitary left kidney is rare occurrence. Though there is no etiological association between Extra Hepatic Portal Vein Obstruction and solitary left kidney but the solitary left kidney decides the modality of treatment. Eighteen year lady referred to our institute with menorrhagia for 5 years and ultrasonography finding of splenomegaly and atretic right kidney. Investigations revealed Extra Hepatic Portal Vein Obstruction with multiple cavernoma formation with oesophagogastric varices with right renal agenesis. She successfully underwent splenectomy with devascularisation. Patient with Extra Hepatic Portal Vein Obstruction present mainly with recurrent episodes of variceal bleeding, splenomegaly and hypersplenism. Splenectomy and esophagogastric devascularisation is an effective modality of treatment for patient with Extra Hepatic Portal Vein Obstruction with solitary kidney.

## INTRODUCTION

Extra hepatic portal vein obstruction (EHPVO) is a vascular disorder which results in obstruction and cavernomatous transformation of portal vein with or without involvement of intrahepatic portal vein, splenic vein or superior mesenteric vein.^1^ EHPVO is important cause of noncirrhotic portal hypertension. Common causes of EHPVO in paediatric population includes neonatal umbilical sepsis, intraluminal trauma during exchange transfusion and pylephlebitis following intraabdominal sepsis.^2^ In adults, EHPVO is rare and common causes includes hypercoaguable states, trauma, myeloproliferative diseases and invasion by tumors.^2,3^ In majority of cases the cause remains unidentified.^2,3^

Most common clinical presentation is upper gastrointestinal bleeding, incidental finding of splenomegaly, poor growth in cases of children and bleeding tendencies.^2^ If left untreated mortality may occur due to bleeding tendencies and infections.^2,3^

## CASE REPORT

Eighteen year female with complains of menorrhagia for 5 years was referred to our center with ultrasonography finding of splenomegaly. There was no history of other bleeding tendencies and pain abdomen. On examination, vitals were stable, pallor was present with no icterus and lymphadenopathy. Per vaginal and per speculum findings were normal. Per abdominal examination revealed massive firm and non-tender splenomegaly measuring 8 cm below the costal margin. No other significant past medical or surgical history. No known evidence of solitary kidney in family.

Blood investigations revealed haemoglobin 8.2 gm/dl and PCV 27.7%, platelets 8000/cumm, total leukocyte count 1,040/cumm with neutrophils 68%, lymphocytes 23%, monocyte 3%. Renal function tests was normal with urea 3 mmol/l and creatinine 62 /vmol/l. Liver function test was normal.

Ultrasonography revealed splenomegaly and upper GI endoscopy revealed two columns of grade II to III esophageal varices and gastroesophageal varices type I with portal hypertensive gastropathy.

**Figure 1.**
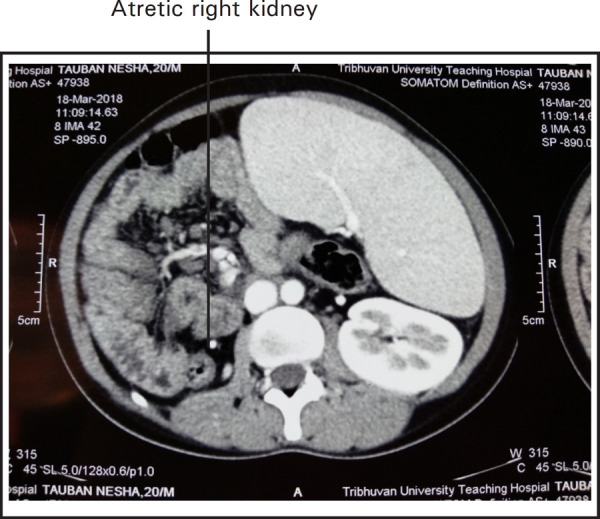
CT scan showing solitary left kidney with splenomegaly.

**Figure 2.**
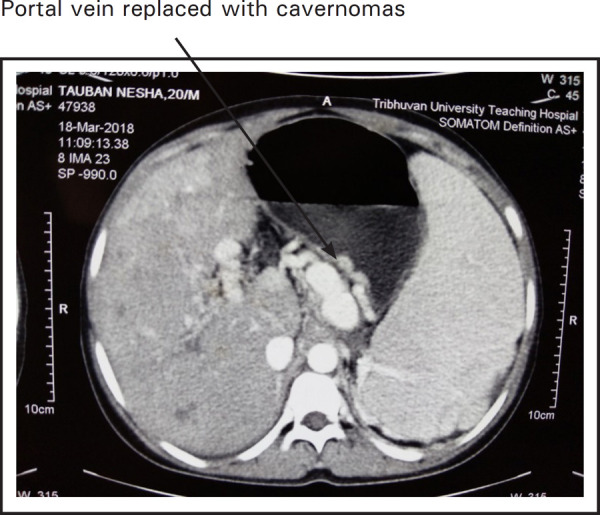
CT scan showing cavernomas and varices.

**Figure 3.**
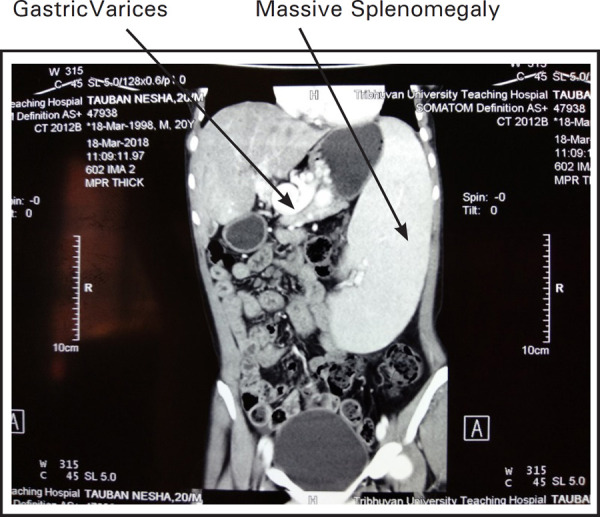
CT scan showing massive splenomegaly with cavernoma and varices.

Contrast enhanced CT scan of abdomen revealed non-visusalization of portal vein replaced with cavernoma formation (Figure 2) along with gastric, esophageal, peripancreatic and pericholecystic varices (Figure 1, 2 and 3) and gross splenomegaly (Figure 3) with atretic right kidney (Figure 1).

Despite shunt surgery with spleen preservation being the management option for the case of EHPVO, splenectomy and esophagogastric devascularisation (modified Hassab's operation) was planned for this patient due to presence of solitary left kidney and completed successfully (Figure 3).

Preoperatively patient was vaccinated against Haemophilus influenza, Neisseria meningitides and Streptococcus pneumoniae. Preoperative findings were hugely enlarged and congested spleen with multiple cavernoma formation.

Postoperative period was uneventful except for significant rise in platelet count with the level reaching more than 10 lakhs on 10^th^ postoperative day then aspirin was started. During routine follow up after a week, platelet count was normal. On monthly follow up till three months, patient was symptomatically better with normal menstrual cycle without menorrhagia. Blood counts and renal function tests were within normal range.

## DISCUSSION

Incidence of unilateral renal agenesis is 1 in 2000. Unilateral renal agenesis may be an isolated congenital abnormality or may be associated with chromosomal or non-chromosomal abnormalities like VACTERL (Vertebral defects, Anal atresia, Cardiac defects, Renal anomalies and limb defects) and MURCS (Mullerian, Renal, Cervicothoracic and Somite).^6^ However, no association has been found between renal agenesis and EHPVO.^7^

Management of EHPVO includes medical, endoscopic and surgical procedure. Medical and endoscopic management includes either prophylaxis or treatment of variceal bleeding. Surgical management includes esophagogastric devascularisation with or without splenectomy and porto-systemic shunt surgeries.^4^

Outcome of splenectomy and esophagogastric devascularisation in case of EHPVO is found to be good. Esophageal varices has been found to be resolved in 62% cases, gastric varices in 100% cases after splenectomy and esophagogastric devascularisation. No surgical mortality has been found to be associated with this procedure. Incidence of variceal bleeding following devascularisation has been found to be 11.5%.^5^

In our case, the patient was eighteen year lady with hypersplenism, gastro-oesophageal varices, replacement of portal vein with cavernomas and solitary left kidney. We performed modified Hassab's operation (splenectomy and gastroesophageal devascularisation) following which menorrhagia improved and hypersplenism was reversed with normal platelet, RBC and WBC counts.

## Consent

**JNMA Case Report Consent Form** was signed by the patient and the original is attached with the patient's chart.

## Conflict of Interest

**None.**
